# Intussusception as the Primary Presentation of a Cecal Tumor

**DOI:** 10.7759/cureus.95501

**Published:** 2025-10-27

**Authors:** Maria Ana Mirante, Firmo Mineiro

**Affiliations:** 1 General Surgery, Unidade Local de Saúde do Médio Tejo, Tomar, PRT

**Keywords:** case report, cecum, colon, intestinal obstruction, intussusception, neoplasia, tumor

## Abstract

We report the case of a 74-year-old female patient who had experienced intermittent abdominal pain over the previous three months. She presented to the emergency department with worsening symptoms and was admitted with a diagnosis of intestinal obstruction. Imaging revealed a colocolic intussusception, with a cecal mass serving as the lead point. Considering the possibility of malignancy, the patient underwent a right hemicolectomy, during which the intussuscepted segment was excised. Pathologic examination confirmed a cecal mucinous adenocarcinoma. Fourteen lymph nodes were retrieved in the surgical specimen, with no evidence of metastatic disease. The patient was placed on an active surveillance regimen, including clinical examinations every three months, thorax, abdominal, and pelvic CT scans at six months, and a total colonoscopy within the first postoperative year.

## Introduction

Intussusception is defined as the invagination of a proximal intestinal segment (the intussusceptum) into an adjacent distal segment (the intussuscipiens). If left untreated, it can progress to intestinal obstruction and vascular compromise of the affected bowel, potentially resulting in necrosis and perforation [1]. In the pediatric population, intussusception is a common abdominal emergency, typically primary (previously termed idiopathic) and often associated with infectious or anatomical predisposing factors [1].

In contrast, adult intussusception is rare, accounting for only 1-5% of all intestinal obstructions and approximately 5% of all intussusceptions [2,3]. A pathological lead point is identifiable in up to 90% of adult cases, most frequently a benign or malignant neoplasm [2-4]. Unlike in children, the classic triad of cramping abdominal pain, bloody diarrhea, and a palpable mass occurs in fewer than 10% of adult patients [2,5]. Adults typically present with chronic, intermittent, and nonspecific symptoms, such as vague abdominal pain, nausea, vomiting, altered bowel habits, or weight loss, which often mimic other gastrointestinal disorders [2,4,5]. Acute presentations with overt obstruction are less common, contributing to delayed or missed diagnoses.

Intussusception can occur at various anatomical sites, including enteroenteric (small bowel), colocolic (large bowel), ileocolic (terminal ileum into ascending colon), and ileocecal (with the ileocecal valve as the lead point) [5]. Among these, ileocolic intussusception is most common, while colocolic involvement is rare, representing a small minority of adult cases [5-7]. When colocolic intussusception does occur, it is frequently associated with malignant lesions of the colon, most commonly adenocarcinoma of the cecum or sigmoid [6-8].

In this context, our patient’s presentation is unusual: colocolic intussusception is an uncommon subtype, and the lead point was a mucinous adenocarcinoma of the cecum, a relatively rare histologic variant. This case underscores the diagnostic challenges posed by atypical and nonspecific symptoms and highlights the importance of early cross-sectional imaging to guide timely surgical management.

## Case presentation

A 74-year-old female patient, with a surgical history of hysterectomy with bilateral oophorectomy and cholecystectomy, presented to the emergency room with a three-month history of epigastric pain radiating to the lower abdomen, mainly the hypogastrium. Over the course of seven days, her pain progressively worsened and was accompanied by intermittent episodes of vomiting, anorexia, and diarrhea.

On physical examination, the patient was fully active and oriented, without neurological deficits, and hemodynamically stable. Abdominal examination revealed hypoactive intestinal sounds and tenderness predominantly in the hypogastrium and right iliac fossa. At the transition between these regions, a palpable, painful, round mass with a seemingly regular surface, approximately 10 cm in diameter, was noted. No palpable lymph nodes were present, and Blumberg’s sign was negative, indicating the absence of peritoneal irritation. No other relevant abnormalities were observed on examination.

**Figure 1:**
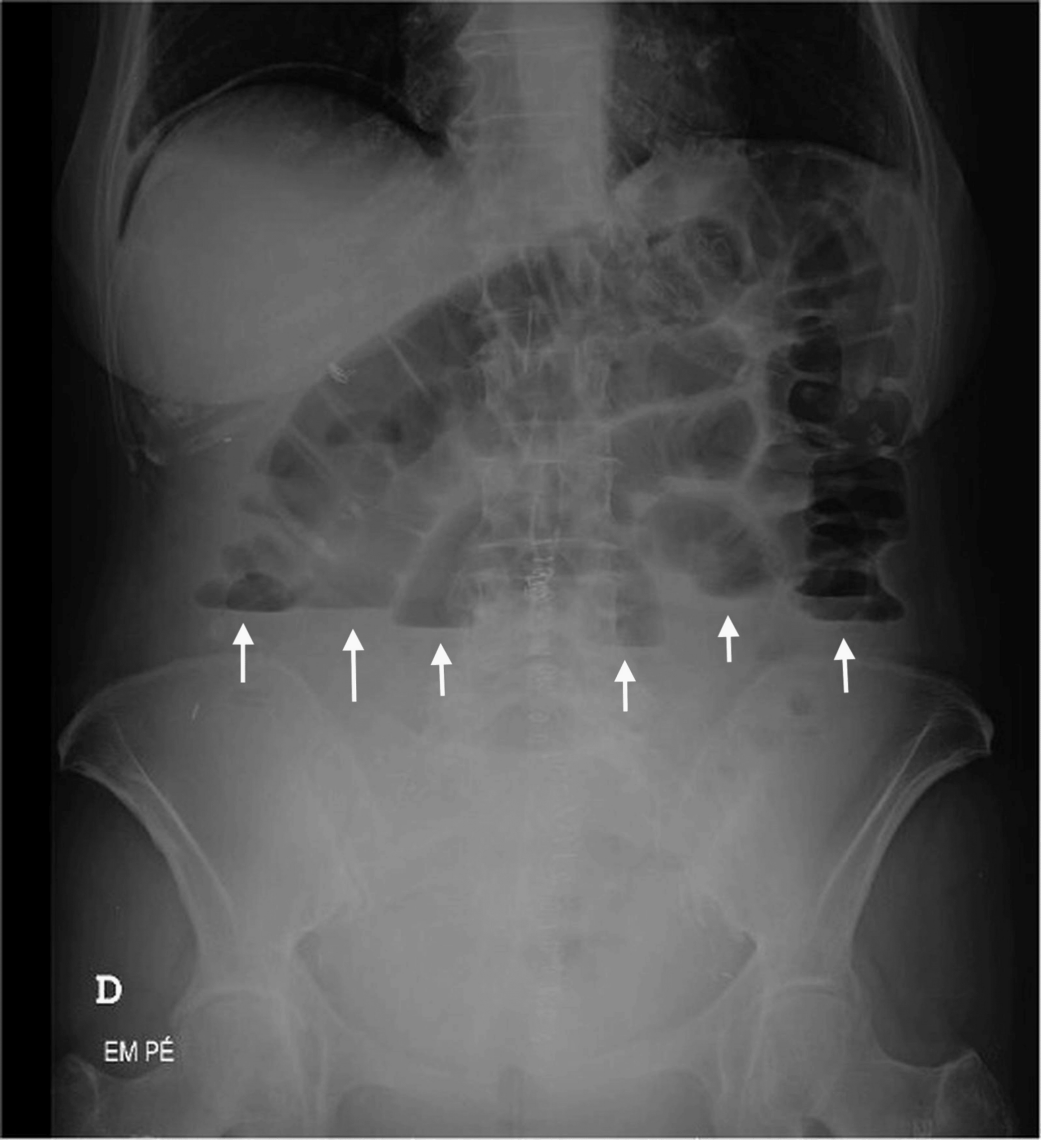
Upright abdominal X-ray demonstrating small-bowel air-fluid levels (arrows)

**Figure 2:**
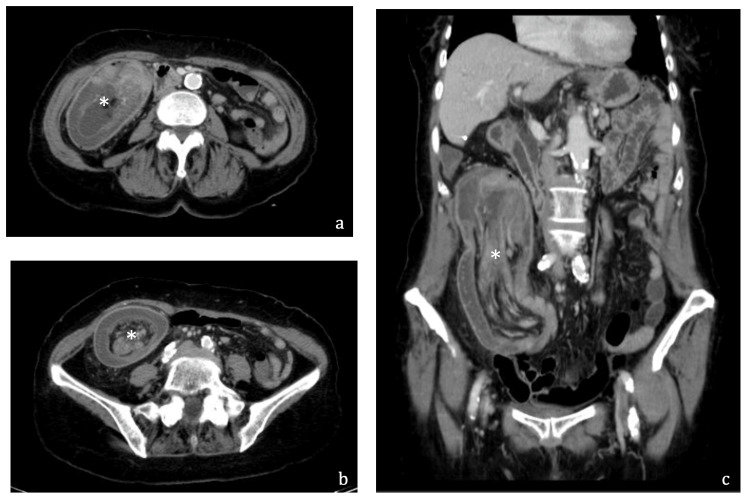
Abdominal and pelvic CT scan in transverse (a, b) and sagittal (c) views demonstrating intussusception of the right colon (asterisk)

Laboratory tests showed normal hemoglobin, no significant elevation of inflammatory markers, and a CRP of 1.89 mg/dL. An abdominal X-ray revealed small bowel air-fluid levels and the absence of air in the colon (Figure 1). Given the lack of an obvious cause for intestinal obstruction, an abdominal and pelvic CT scan was performed (Figure 2).

**Figure 3:**
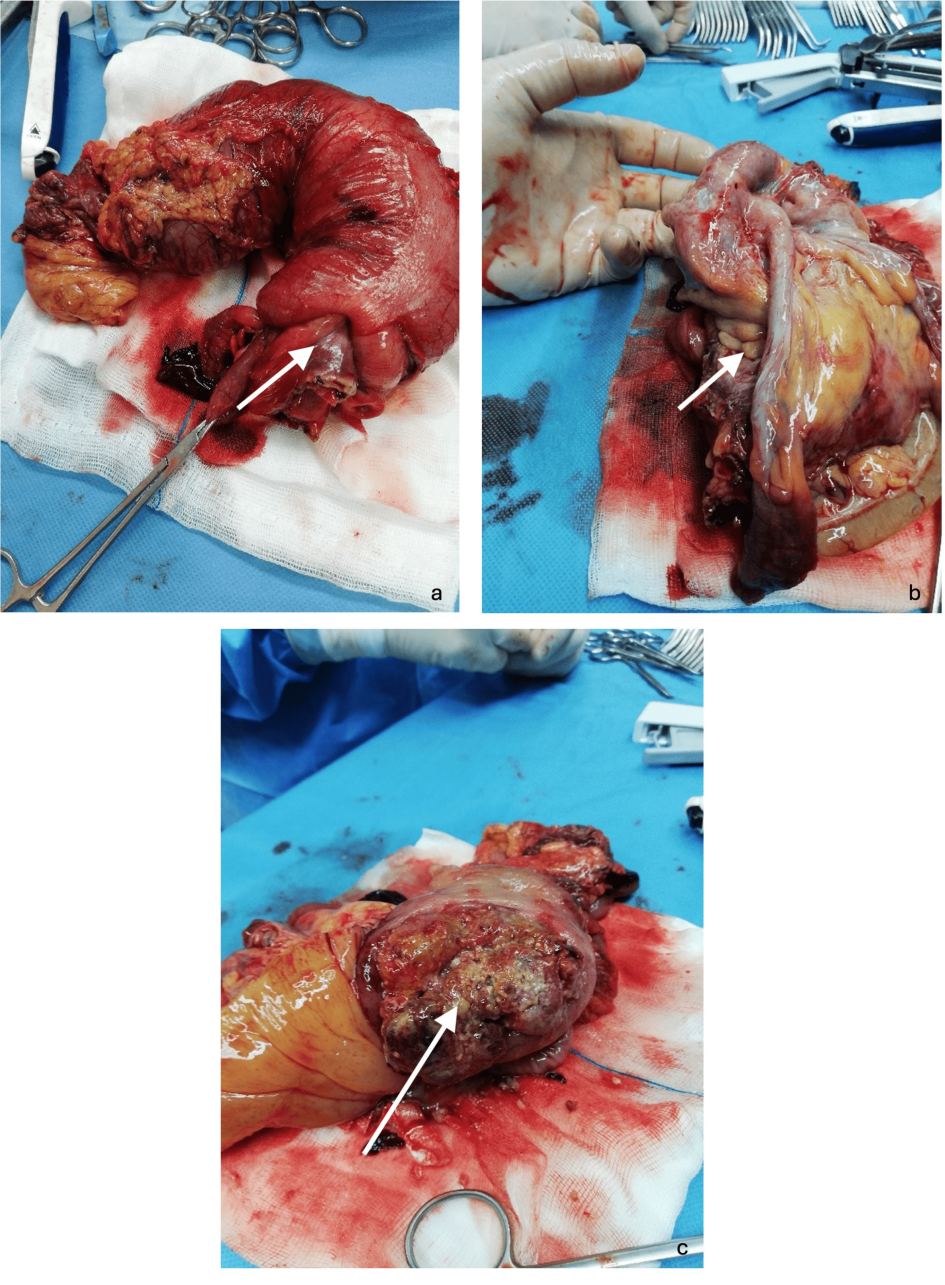
Intraoperative findings: (a) intussusception point (arrow), (b) appendix (arrow), and (c) lead point of the intussusception after opening the specimen (arrow)

The abdominal and pelvic CT scan revealed a cecal intussusception extending approximately 16 cm, with a characteristic “whirl” of intestinal loops at the head of the intussuscepted segment, measuring roughly 63 × 45 mm, and involving fat and mesenteric vessels. The biliary tract appeared dilated, likely due to compression of the pancreas and duodenum by the intussuscepted bowel. The patient consented to an exploratory laparotomy, which was performed via a midline incision. Intraoperatively, the cecum was found to be intussuscepted, with pronounced proximal bowel dilation and multiple serial tears. Given the high likelihood of an underlying malignancy, a right hemicolectomy was performed. Gross examination of the excised segment identified the point of intussusception, a free appendix, and an extensive cecal tumor, confirming a colocolic intussusception in which the cecum had invaginated through the ascending colon, with the tumor serving as the lead point (Figure 3).

The postoperative period was uneventful. The patient began oral intake two days after surgery, resumed bowel function by day six, and was discharged on postoperative day nine. Pathology revealed an invasive cecal mucinous adenocarcinoma with lymphovascular and perineural invasion, involving 14 lymph nodes without metastatic disease (pT3N0, dMMR - MSI). After multidisciplinary discussion, it was decided to pursue clinical surveillance due to the patient’s age, MSI status, and the favorable prognosis associated with stage II disease.

## Discussion

Adult intussusception is uncommon and diagnostically challenging, as symptoms are often chronic, intermittent, and nonspecific, in contrast to the typically acute and benign pediatric presentation [1,2]. In adults, an underlying structural lesion can be identified in the majority of cases, and when the colon is involved, a malignant neoplasm is frequently the lead point [2,4-6]. Although most adult cases present with chronic or intermittent symptoms, acute abdominal pain mimicking other surgical emergencies has also been reported [9]. Our patient’s subacute course, with obstructive symptoms, CT demonstration of a “target/sausage” configuration, and operative confirmation of a colocolic intussusception with a cecal mass as the lead point, exemplifies these patterns.

Epidemiologically, most adult intussusceptions are enteroenteric; however, colonic involvement, particularly ileocolic/ileocecal, carries the highest likelihood of malignancy, with several series and reviews reporting carcinoma as the lead point in a substantial proportion of colonic cases [4-6]. This aligns with our finding of a cecal adenocarcinoma as the etiology. Prior reports specifically highlight cecal/ileocecal primaries as common causes of malignant adult intussusception, reinforcing the need for oncologic vigilance when imaging localizes the process to the right colon [6-8].

Imaging plays a central role in diagnosis and localization. While plain radiography may only suggest obstruction, ultrasonography can demonstrate the “target” sign, particularly in pediatric cases. Earlier reports have detailed the typical endoscopic and radiologic findings associated with cecal intussusception [10]. Endoscopic visualization can occasionally reveal the lead-point lesion, such as a cecal mass causing intussusception [11]. In adults, contrast-enhanced CT is considered the most sensitive and specific modality, defining location, configuration, and often suggesting a lead point [1,5]. In our case, CT not only established the diagnosis but also raised suspicion for a mass, guiding early surgical decision-making consistent with contemporary recommendations [1,5].

Transient, self-limiting enteroenteric intussusceptions, occasionally observed during normal peristalsis, have been described in the literature. These typically involve short, non-obstructing segments (<3.5 cm) that resolve spontaneously and lack features of obstruction or a lead point, distinguishing them from clinically significant intussusceptions requiring intervention [1].

Management strategies differ markedly between children and adults. Nonoperative reduction (air or hydrostatic enema) is standard in pediatrics due to the usually benign etiology. In adults, especially with colonic involvement, primary surgical resection is widely recommended because of (i) the high incidence of pathologic lead points; (ii) the risk of ischemia or perforation; and (iii) oncologic concerns regarding intraluminal tumor seeding with attempted reduction [1,2,4-6]. Consistent with these principles, we performed a right hemicolectomy without prior reduction, achieving both diagnosis and treatment in a single oncologic operation.

The choice of operative approach can be individualized. Several reports describe successful laparoscopic management, sometimes after cautious preoperative reduction, when malignancy risk is low or the lead point is uncertain [12]. Nevertheless, for right-sided colonic intussusceptions where carcinoma is suspected, en bloc resection with oncologic technique remains standard, and open surgery is often preferred if there is significant distension, friability, or concern for perforation [4-6,13]. Our intraoperative findings of edema and serosal tears favored a definitive resection-first strategy.

Histologically, our patient had a mucinous cecal adenocarcinoma (pT3N0). Mucinous histology has been reported as a lead point in adult colonic intussusception and may have distinct biological behavior and prognostic implications compared with non-mucinous adenocarcinoma, although outcomes ultimately depend on stage and nodal status [7,8,13]. Our patient had negative lymph nodes and early intervention, both of which are associated with favorable short-term outcomes, with five-year overall survival reported around 86% after curative resection (five-year mortality ~14%) [14-16].

Endoscopic evaluation has a limited but complementary role in adults. Colonoscopy may help localize the lesion and obtain tissue in cases of partial obstruction, but it must be approached cautiously due to the risk of perforation in a distended, edematous colon. In suspected malignant colocolic intussusception, preoperative colonoscopy rarely alters the need for oncologic resection [1,5]. In our case, cross-sectional imaging directed expedited surgery.

In summary, our case underscores several literature-supported principles: (i) adult colonic intussusception is uncommon but often associated with malignancy; (ii) CT provides decisive diagnostic and anatomic information; and (iii) oncologic resection without attempted reduction is usually appropriate when a colonic primary is suspected. The identification of a mucinous cecal adenocarcinoma as the lead point is consistent with prior reports, and the pT3N0 outcome supports the benefit of prompt surgical management [1,4-8,13].

## Conclusions

In adults, an underlying structural lesion is identified in the vast majority of intussusception cases, with malignancy being a leading cause, particularly when the colon is involved. Several large reviews indicate that up to 65-70% of adult colonic intussusceptions are secondary to malignant neoplasms, most commonly adenocarcinomas of the cecum or sigmoid colon. In contrast, small-bowel intussusceptions are more frequently caused by benign lesions such as lipomas, polyps, or Meckel’s diverticulum. The high prevalence of malignancy justifies the principle that adult intussusception should be considered malignant until proven otherwise. Chronic, nonspecific symptoms and often subacute evolution can delay diagnosis, increasing the risk of ischemia or perforation. Therefore, once identified on imaging, especially when the colon is involved, oncologic surgical resection without prior reduction remains the standard of care.

Our patient exemplifies this paradigm: CT findings of colocolic intussusception with a lead-point mass raised strong suspicion for malignancy, prompting early surgical intervention. Histopathological analysis confirmed a mucinous adenocarcinoma of the cecum (pT3N0). Although mucinous adenocarcinoma is less common than conventional adenocarcinoma, it is well recognized as a potential lead point in malignant intussusception. While this histologic subtype has distinct biological behavior, prognosis in stage II disease remains favorable, with an estimated 5-year overall survival of approximately 86% following curative resection. Collectively, these findings reinforce that adult colonic intussusception should always prompt oncologic vigilance and that early surgical management guided by imaging is both diagnostic and therapeutic, preventing complications and ensuring adequate oncologic margins.
